# Association of mitochondrial respiratory chain enzyme activities with embryo transfer success

**DOI:** 10.3168/jdsc.2026-1078

**Published:** 2026-08-06

**Authors:** Ashley Waymire, Ashley M. Niesen, Heidi A. Rossow

**Affiliations:** Veterinary Medicine Teaching and Research Center School of Veterinary Medicine, University of California Davis, Tulare, CA 93274

## Abstract

Mitochondria produce energy vital for health, reproduction, and metabolism and contain their own DNA, which is inherited exclusively from the dam. The objective of this study was to assess the association of mitochondrial enzyme activities of Complex I (C-I), Complex IV (C-IV), and Complex V (C-V) with pregnancy outcome and other physiological and management factors influencing reproductive efficiency. Forty Holstein recipients were assigned either embryo A (Donor 1/Sire 1, n = 20) or embryo B (Donor 2/Sire 2, n = 20) based on donor embryo production and recipient availability. Crude mitochondrial extracts were isolated from peripheral blood mononuclear cells and analyzed for mitochondrial enzyme activities of C-I, C-IV, and C-V. Pregnancy outcome was checked at ∼32 d carried calf, confirmed at 45 and 90 d carried calf, and by live calf birth. Mitochondrial enzyme activities were regressed on management, genomic, embryo source, and pregnancy outcome and confirmed through birth. Performance data were collected from the herd management software DairyComp 305 (VAS, Tulare, CA), and hematological variables were measured at the time of embryo transfer. The mitochondrial enzyme activity of C-I was associated with C-IV, C-V, and mean corpuscular hemoglobin. Complex IV was associated with C-I, C-V, and platelets, and C-V was associated with C-I, C-IV, lymphocyte number, and genomic Dairy Wellness Profit Dollars (Zoetis). Embryo source also tended to influence C-I and C-IV activities. This effect was replaced with genomic daughter pregnancy rate when embryo was not included in the regressions. Associations between C-I and C-IV reflect their joint role in creating the proton gradient in the electron transport chain and relationship with oxygen supply and immune function. Mitochondrial enzyme activities were shaped by genetic and physiological factors but, in this study, were not a definitive marker for reproductive success of recipients via embryo transfer. But further examination of the relationship between mitochondrial function and nuclear-encoded genes to find markers of success of pregnancy via embryo transfer is warranted.

## Graphical Abstract

**Figure:**
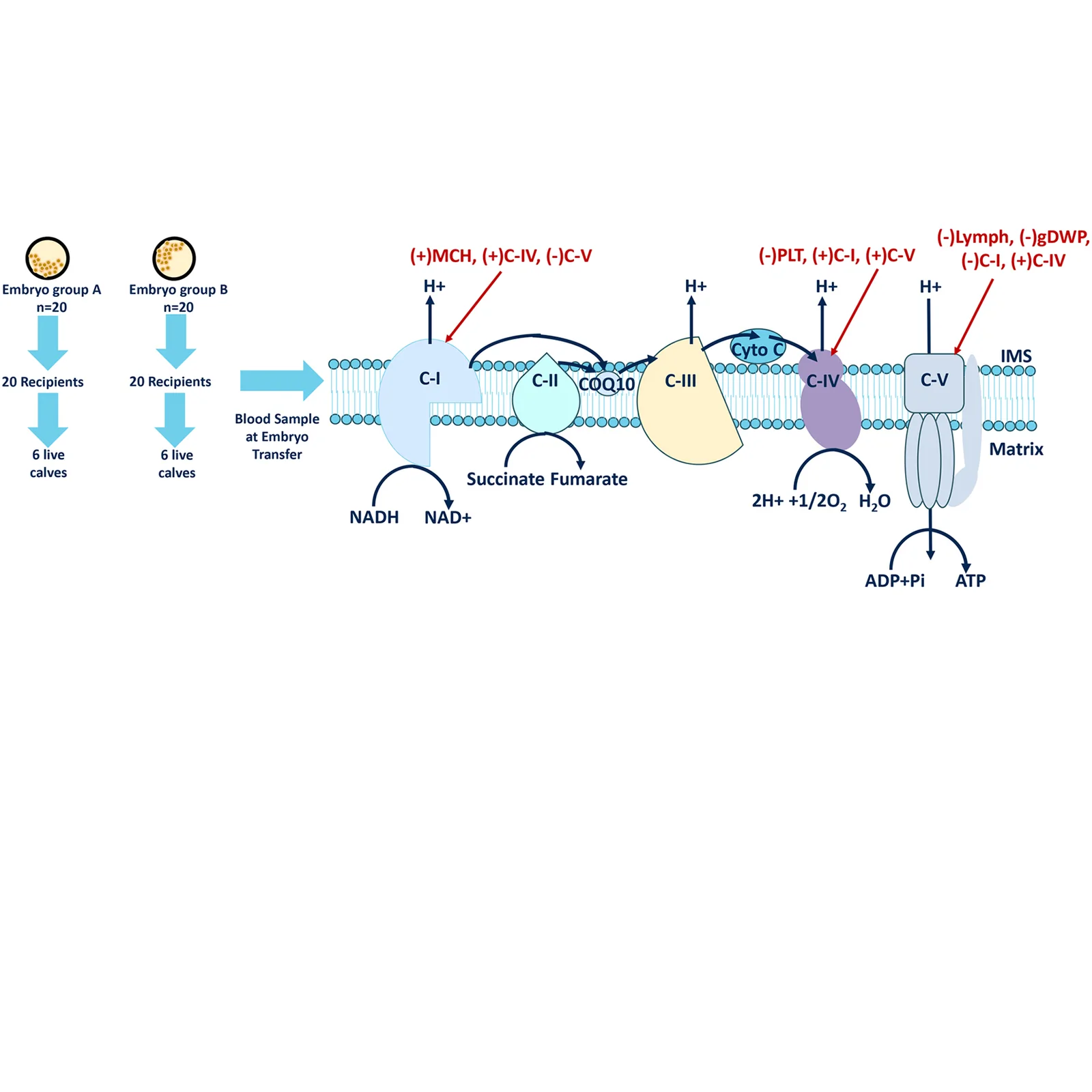

Mitochondria generate the energy essential for metabolism, production, health, and reproduction and possess their own DNA. Despite this, genetic progress and selection in dairy cattle breeding have traditionally focused on paternal traits, overlooking maternal contributions. With the growing use of beef semen and a shrinking domestic heifer inventory, selecting the best females for the next generation has become more critical. The dairy industry has advanced in identifying top-ranking females for sexed semen allocation, but less attention has been given to those in lower percentiles who are commonly designated for beef semen or serve as embryo transfer recipients. Reproductive technologies such as embryo transfer enable the production of high-value, genetically superior animals from lower-value recipient animals. Recipient selection typically relies heavily on genetic merit, placing these females in a lower-ranking group. Within this group, limited attention is given to identifying the most suitable candidates, which can affect the success of these costly pregnancies. Exploring the contribution of mitochondrial function to reproductive outcomes could provide an opportunity to maximize economic inputs for breeding and selection.

Mitochondria are of interest in evaluating reproductive success because of their role in cellular energy metabolism. In reproduction performance, most mitochondrial research has focused on mitochondrial count and ATP in oocytes as impacted by the age of the donor or season. Research in humans (Bentov et al., 2011) and cattle (Iwata et al., 2011; Kansaku et al., 2017) have shown that mitochondrial count and ATP content positively impact the competency of oocytes and the outcomes of in vitro embryos. Younger animals have oocytes with higher mitochondrial counts and ATP compared with older animals, resulting in greater reproductive success. To date, no studies have correlated dairy cattle reproductive outcomes with mitochondrial enzyme activities (**MEA**), which are instrumental in ATP production.

The effect of maternal lineage has provided insights on correlations with performance. However, mitochondrial processes rely on proteins encoded by both nuclear and mitochondrial DNA, so further investigation of performance with mitochondrial processes and function is needed.

The hypothesis of this study is respiratory chain enzyme activities, including Complex I (**C-I**), Complex IV (**C-IV**), and Complex V (**C-V**) in peripheral blood mononuclear cells (**PBMC**) at the time of embryo transfer, affect pregnancy outcome. The objective of this study was to determine if MEA of C-I, C-IV, and C-V is associated with pregnancy outcome and other physiological and management factors influencing reproduction.

A prospective observational study comparing MEA in recipients with embryo transfer pregnancy outcomes was conducted using 40 mostly primiparous lactating Holstein cows serving as embryo recipients (n = 40) between 70 and 120 DIM. They were selected by the farm based on their genetic merit percentile within the herd, the number of eligible females available for breeding for the month, and the number of heifer calf pregnancies required for the month from a commercial dairy herd of ∼2,800 cows located in southern Wisconsin.

Virgin yearling Holstein heifers (n = 2) were used as oocyte donors for in vitro production of embryos. Recipients received 1 of 2 embryo matings (A or B) at 70 to 120 DIM, with 20 recipients in group A and 20 in group B. A minimum sample size of 12 recipients per embryo mating was determined based on a 2-tailed test with a difference of 50% between electron transport chain enzyme complex activities of the recipient cow or heifer with a power of 0.80 and an α of 0.05 (Niesen et al., 2022). Records were entered by farm personnel into a dairy management software program (DairyComp305, VAS, Tulare, CA). Genomic data were collected on all donor and recipient cows enrolled.

Two nulliparous donors were collected on a biweekly (every 2 wk) basis in alternating weeks for in vitro embryo production. Before ovum pickup (**OPU**), donor heifers underwent follicle stimulation. Donors administered were a 2 mL dose of Gonadotropin Releasing Hormone (GnRH; Fertagyl, Merck Animal Health, Rahway, NJ) 6 d before OPU, followed 48 h later by 6 follicle stimulating hormone (FOLLTROPIN, Vetoquinol USA, Fort Worth, TX) injections. The FSH doses were administered in decreasing dosages, concluding 24 h before OPU. For the OPU procedure, the 2 donor heifers were restrained in a squeeze chute and provided epidural anesthesia (5 mL of 2% lidocaine hydrochloride; Clipper Distributing Company, St. Joseph, MO). Follicular aspiration was conducted using a transvaginal ultrasound-guided technique with the Evo III ultrasound (IBEX, E.I. Medical Imaging, Loveland, CO) and an OPU ultrasound probe (E.I. Medical Imaging, Loveland, CO, eC9 OPU) for cattle. Follicles greater than 3 mm in diameter were aspirated using a 17-gauge, 600-mm needle. The needle was connected via plastic tubing to a 50-mL conical tube with 5 mL of heparinized complete flush solution (ABT Complete Flush, ABT 360, Pullman, WA). The conical tube was held in a tube warmer maintained at 36.5°C and was also separately linked to a vacuum pump consistently operated at 25 mL/min. The resulting follicular content was filtered through a 100-μm cell strainer and rinsed into a warmed grid-searching dish with complete flush solution. Cumulus oocyte complexes were located using a stereomicroscope. In vitro production methods were followed as described in Brown et al. (2024). Embryos in stages 6 to 9 and grade 1 per International Embryo Transfer Society guidelines (Stringfellow and Seidel, 2010) were used for embryo transfer. Each embryo was loaded with holding medium into a 0.25-mL French straw (IMV Technologies, L'aigle, Basse-Normandie, France), placed into a portable straw incubator set at 36.5°C, and transported to the farm for fresh embryo transfer with a holding time no greater than 3 h.

Recipients were synchronized using a timed double-ovsynch protocol 34 d before embryo transfer. Estrus was not observed; cows with a palpable corpus luteum at time of transfer, had an embryo transferred into the ipsilateral uterine horn and received 2.5 mL of human chorionic gonadotropin (Chorulon, Merck Animal Health, Rahway, NJ) intramuscularly. Pregnancy was diagnosed via transrectal ultrasound at ∼32, 45, and 88 d carried calf (**DCC**) and by palpation at 200 DCC. Because embryos were ∼7 d at embryo transfer, the initial pregnancy check occurred around 25 d post embryo transfer. In this context, DCC represents embryonic age in a successfully maintained pregnancy. Calving ease was recorded and scored on a scale of 1 to 5 (1 = no problems encountered or unobserved birth; 5 = extreme difficulty) consistent with the National Association of Animal Breeders and USDA program for calving ease evaluations (Berger, 1994). Live and stillborn calves were recorded in the herd management software along with birth weight and sex.

Whole blood samples were obtained via coccygeal venipuncture at the time of embryo transfer from recipients. Blood samples were collected into tubes containing K2 EDTA anticoagulant (BD Biosciences). Blood samples were refrigerated, shipped overnight on ice, and processed within 24 h of collection.

Hematology analysis was performed on whole blood samples (Drew Scientific Hemavet 950 Hematology Analyzer from Erba Diagnostics, Oxford, CT) to evaluate the immune status of recipients. Then mitochondria were isolated from the PBMC fraction and isolated using the Mitochondrial Isolation Kit for Cultured Cells (Abcam, Cambridge, MA; ab110170) according to the manufacturer's protocol and as detailed by Niesen et al. (2022).

Mitochondrial enzymatic activity assays were conducted following the collection and processing of all samples into crude mitochondrial extracts. Mitochondrial protein content in the crude extracts was quantified using a Bicinchoninic acid protein assay (Abcam, Cambridge, MA; ab102536) according to the manufacturer's protocol, allowing enzyme activities to be expressed relative to mitochondrial protein content. Enzyme activity was measured using microplate assay kits (Abcam, Cambridge, MA) following the manufacturer's protocol and as detailed by Niesen et al. (2022). All kits were bovine species reactive and from the same lot. Negative background controls were prepared using the buffer solution provided in the enzyme kits, whereas positive controls consisted of bovine heart mitochondria (Abcam, Cambridge, MA; ab110338) standardized to 5.5 mg/mL of total protein. The microplates were incubated for 3 h at room temperature before absorbance measurements were taken using a VersaMax tunable microplate spectrophotometer (Molecular Devices, Sunnyvale, CA) in kinetic mode. Readings were taken every 30 to 60 s for 1 h, depending on the enzyme kit.

Cow was the experimental unit of interest. Difference was determined by *P* ≤ 0.05, with tendencies considered as 0.05 < *P* ≤ 0.10. All residuals were examined for normality. All data were expressed as LSM. Backward selection was used to remove independent variables that did not contribute to the regression analysis. Residuals for all regressions were normally distributed.

Enzyme activity was defined as the linear rate of change of absorbance per min, adjusted to the micrograms of crude mitochondrial protein in the well (milli optical density units/min per µg mitochondrial protein). For each sample, absorbance data time points were used to calculate a single slope, which represented the enzyme activity value for that animal. The slope (enzyme activity) was estimated using general linear models procedure to regress optical density on time (SAS 9.4, SAS Institute Inc., 2024, Cary, NC). Outliers beyond 2 SD were excluded and final activities were corrected by the amount of crude mitochondrial protein in the sample. The model used in analysis was Y_OD_ = β_0_ + β_1_Time + ε, in which Y_OD_ = optical density, β_0_ = y-intercept, β_1_ = regression coefficient for time, and ε = residual error.

Cow performance, hematology, and MEA analysis were conducted using the general linear models procedure of SAS. To assess initial differences between recipient groups, a model was run for each of the following animal performance metrics as the dependent variable: prewean ADG, heifer days to conception, weight at first calving, age at first calving, 4 wk daily milk average, 8 wk daily milk average, 12 wk daily milk average, genomic Dairy Wellness Profit Dollars (**gDWP**), genomic daughter pregnancy rate (**gDPR**), and genomic cow conception rate (**gCCR**). In each model, embryo, age at transfer, DIM at embryo transfer, and the other performance metrics were included as independent variables in the preliminary model. Each trait was analyzed once as the dependent variable and included as an independent variable in the models for the other traits.

For hematology analysis, each hematological variable was regressed on the independent variables of pregnancy outcome confirmed through 90 DCC, embryo treatment, DIM at enrollment, age at enrollment, transfer date, lactation, embryo transfer technician, service number, calving ease of prior calving, sex of calf of prior calving, and hematological variables. Each hematological variable was included as an independent variable in the preliminary model. Hematology variables were included to allow for a better understanding of the immune state and its potential impact on MEA. Statistical differences within the clinically normal range were defined as not different.

For MEA analysis, MEA for each enzyme was regressed on the independent variables of pregnancy outcome confirmed through 90 DCC, embryo treatment, DIM at enrollment, age at enrollment, transfer date, lactation, embryo transfer technician, service number, calving ease of prior calving, sex of calf of prior calving, other MEA (C-I, C-IV, C-V), hematological variables, and performance variables. These variables were selected due to their connection with reproductive outcomes.

The resulting model for C-I was C-I = β0 + β1 pregnancy outcome + β2 embryo source (A or B) + β3 interaction of pregnancy and embryo source + β4 C-IV + β5 C-V + β5 mean corpuscular hemoglobin (**MCH**) + ε, in which C-I = enzyme activity for Complex I, β0 = intercept, β1 = regression coefficient for pregnancy outcome, β2 = regression coefficient for embryo source, β3 = regression coefficient for interaction between pregnancy and embryo, β4 = regression coefficient for C-IV, β5 = regression coefficient for C-V, β6 = regression coefficient for MCH, and ε = residual error.

The resulting model for C-IV was C-IV = β0 + β1 pregnancy outcome + β2 embryo source (A or B) + β3 interaction of pregnancy and embryo source + β4 C-I + β5 C-V + β5 platelets + ε, in which C-I = enzyme activity for Complex I, β0 = intercept, β1 = regression coefficient for pregnancy outcome, β2 = regression coefficient for embryo source, β3 = regression coefficient for interaction between pregnancy and embryo, β4 = regression coefficient for C-I, β5 = regression coefficient for C-V, β6 = regression coefficient for platelet number, and ε = residual error.

The resulting model for C-V was C-V = β0 + β1 pregnancy outcome + β2 embryo source (A or B) + β3 interaction of pregnancy and embryo source + β4 C-I + β5 C-IV + β6 lymphocyte number + β7 gDWP + ε, in which C-V = enzyme activity for Complex V, β0 = intercept, β1 = regression coefficient for pregnancy outcome, β2 = regression coefficient for embryo source, β3 = regression coefficient for interaction between pregnancy and embryo, β4 = regression coefficient for C-I, β5 = regression coefficient for C-IV, β6 = regression coefficient for lymphocyte number, β7 = regression coefficient for genomic Dairy Wellness Profit Dollars, and ε = residual error.

The corresponding models used when embryo source was removed from the regression equations were C-I = β0 + β1 pregnancy outcome + β2 DIM at transfer + β3 C-IV + β4 age at first calving + β5 weight at first calving + β6 gDPR + ε, in which C-I = enzyme activity for Complex I, β0 = intercept, β1 = regression coefficient for pregnancy outcome, β2 = regression coefficient for recipient DIM at embryo transfer, β3 = regression coefficient for C-IV, β4 = regression coefficient for age at first calving, β5 = weight at first calving, β6 = regression coefficient for genomic Dairy Wellness Profit Dollars, and ε = residual error. The model for C-IV was C-IV = β0 + β1 pregnancy outcome + β2 C-I + β3 gDPR + ε, in which C-IV = enzyme activity for Complex IV, β0 = intercept, β1 = regression coefficient for pregnancy outcome C-I, β2 = regression coefficient for C-I, β3 = regression coefficient for genomic daughter pregnancy rate, and ε = residual error. The model for C-V was exactly the same as in the previous analyses.

To assess potential initial differences in recipient uniformity on PBMC MEA, prior performance variables were compared between recipient groups (Table 1). These variables included pre-wean growth performance such as ADG, parturition metrics such as days to conception, age at first calving, and weight at first calving, early lactation milk production including weekly average for 4, 8, and 12 wk of the current lactation, and gDWP, gDPR, and gCCR. Age and DIM at time of enrollment were also evaluated. No differences were observed among any of the variables between recipient groups, indicating that the quality of recipients was consistent between recipient groups.

**Table 1:** Least squares means comparisons of recipient age and DIM at enrollment and prior performance metrics between recipient groups and pregnancy diagnosis

Metric	Embryo group		Pregnancy diagnosis		SEM	*P*-value^1^	
	Recipients A^2^ (n = 20)	Recipients B^3^ (n = 20)	Pregnant (n = 12)	Nonpregnant (n = 28)		Embryo	Preg
Age at enrollment (d)	860	839	889	810	30	0.47	0.03
DIM at enrollment (d)	96	94	98	92	2.5	0.30	0.03
ADG to weaning (kg/d)	0.84	0.82	0.84	0.83	0.014	0.16	0.66
Age at first calving (d)	700	700	700	700	1.23	0.87	0.78
Weight at first calving (kg)	644	648	652	639	7.2	0.58	0.18
4 wk milk avg^4^ (kg)	40.1	39.8	40.0	39.9	0.54	0.54	0.93
8 wk milk avg (kg)	43.2	43.5	43.2	43.5	0.43	0.42	0.71
12 wk milk avg (kg)	43.1	42.5	42.9	42.8	0.62	0.32	0.93
gDWP^5^	771	806	762	815	46	0.45	0.31
gDPR^6^	−0.8	−0.8	−0.8	−0.7	0.14	0.93	0.53
gCCR^7^	0.4	0.4	0.4	0.4	0.17	0.59	0.97

1 Embryo: *P*-values for differences due to embryo A or embryo B; Preg: differences due to pregnancy or nonpregnancy.

2 Cows that received embryo A.

3 Cows that received embryo B.

4 Average milk per day estimated from DairyComp.

5 August 2024 genomic value for Zoetis Dairy Wellness Profit Dollars.

6 August 2024 genomic value for daughter pregnancy rate trait.

7 August 2024 genomic value for cow conception rate trait.

Of the 40 recipients, 12 conceived: 6 received embryo A and 6 received embryo B. All 12 recipients remained pregnant from the initial pregnancy diagnosis at 32 DCC through 90 DCC. Our power calculation was based on 8 pregnancies for each embryo group, which leaves our analyses underpowered and may have led to the lack of difference (or tendency) to detect a difference in C-I, C-IV, and C-V MEA between embryo groups.

A precise genetic strategy that calculated the number of heifers required each month and strategically ranked breeding-eligible animals monthly on the gDWP selection index was used on this herd (Table 1). High-ranking females received sexed semen to create the next generation of replacements, and lower-ranking females were assigned to beef semen, embryo transfer, or, in a few cases, conventional Holstein semen. Compared with their herd mates of the same age and lactation stage who were not in the trial, enrolled females averaged over 60 points lower on gDWP, were the same for gDPR and gCCR, and had less than 0.5 kg variation in milk yield at 4, 8, and 12 wk of lactation. Enrolled cows and heifers calved 35 d earlier and weighed 9.55 kg less at first calving compared with their herd mates. Despite their lower genetic merit, which categorized them as recipients, enrolled heifers required 0.5 fewer services per conception compared with their herd mates, resulting in earlier conception and explaining their earlier age and lighter weight at calving. LSM performance metrics were also compared between recipient groups by pregnancy diagnosis confirmed through 90 DCC (Table 1). Age and DIM at enrollment were different between pregnant and nonpregnant animals. Of the 40 cows enrolled, 37 were first lactation, 2 were second lactation, and 1 was third lactation. Parity did not make a difference in any of the results in the analyses of other management variables, hematology variables (Table 2), or MEA (Table 3). But because the number of multiparous cows was small, this study is unable to determine if parity (primiparous vs. multiparous) could influence pregnancy diagnosis.

**Table 2:** Least squares means hematological variables by pregnancy diagnosis

Variable^1^	Reference range^2^	Pregnancy diagnosis		SEM	*P*-value	
		Pregnant (n = 11)^3^	Nonpregnant (n = 28)		Preg^4^	Embryo A vs. B^4^
Leukocytes						
White blood cells (K/μL)	4.0–12.0	7.25	7.25	0.0042	0.53	0.51
Neutrophils (K/μL)	0.6–4.1	4.32	4.32	0.0021	0.12	0.71
Lymphocytes (K/μL)	2.5–7.5	2.63	2.63	0.0021	0.13	0.70
Monocytes (K/μL)	0.0–1.2	0.183	0.186	0.0020	0.11	0.74
Eosinophils (K/μL)	0.0–2.4	0.086	0.089	0.0020	0.18	0.43
Basophils (K/μL)	0.0–0.4	0.021	0.024	0.0022	0.16	0.94
Erythrocytes						
Red blood cells (M/μL)	5.0–10.0	6.30	6.31	0.0090	0.34	0.27
Hemoglobin (g/dL)	8.0–15.0	9.26	9.26	0.019	0.76	0.81
Hematocrit (%)	24.0–46.0	27.1	27.1	0.036	0.67	0.97
Mean corpuscular volume (fL)	40.0–60.0	43.1	43.1	0.037	0.34	0.01
Mean corpuscular hemoglobin (pg)	11.1–17.0	14.7	14.7	0.026	0.45	0.73
Mean corpuscular hemoglobin concentration (g/dL)	28.2–36.0	34.2	34.2	0.047	0.83	0.14
Red cell distribution width (%)	12.0–27.0	21.5	22.0	0.38	0.25	0.16
Thrombocytes						
Platelets (K/μL)	200–800	304	210	41	0.03	0.79
Mean platelet volume (fL)	5.0–20.0	7.36	7.12	0.35	0.50	0.74

1 M/μL = million cells per microliter; K/μL = thousand cells per microliter.

2 Normal cow hematology reference ranges preset on the Hemavet 950 FS analyzer.

3 One pregnant animal was not included in the analysis due to poor blood sample quality.

4 Preg: *P*-values for differences due to pregnancy or nonpregnancy; Embryo A vs. B: *P*-values for differences due to embryo A versus embryo B.

**Table 3:** Least squares means recipient mitochondrial enzyme activity by pregnancy diagnosis and embryo sire and donor (A or B)

Mitochondrial enzyme^2^	Enzyme activity				SEM	*P*-value^1^									R^2^
	Pregnant (n = 12)	Nonpregnant (n = 28)	Embryo A (n = 20)	Embryo B (n = 20)		Preg	Embryo	Complex I	Complex IV	Complex V	MCH^3^	PLT^3^	Lymph^3^	gDWP^3^	
Complex I	0.0247	0.0171	0.0307	0.0111	0.012	0.4	0.1		<0.0001	0.0006	0.008				0.63
CI^4^	0.0076 to 0.041		−0.0027 to 0.047												
Complex IV	0.273	0.233	0.297	0.209	0.10	0.5	0.1	<0.0001		0.004		0.02			0.61
CI	0.17 to 0.38		0.13 to 0.38												
Complex V	0.0708	0.0710	0.0671	0.0741	0.014	0.9	0.4	0.0007	0.02				0.01	0.05	0.53
CI	0.059 to 0.082		0.058 to 0.084												

1 Only *P-*values for independent variables that are different (*P* ≤ 0.05) are shown. There were no interactions between pregnant, nonpregnant, and sire-dam pairing for embryo A or sire-dam pairing for embryo B (*P* > 0.7, *P* > 0.8, *P* > 0.2 for Complex I, Complex IV, and Complex V activities, respectively).

2 Units of all mitochondrial enzyme activities (Complex I, Complex IV, and Complex V) are milli optical density/min per μg of mitochondrial protein.

3 MCH is mean corpuscular hemoglobin, PLT is platelet count, Lymph is lymphocyte number, gDWP is genomic Dairy Wellness Profit Dollars.

4 CI are 95% CI for Complex I, Complex IV, and Complex V mitochondrial enzyme activities for pregnancy outcome and embryo A or B.

Hematology variables were measured for each recipient using samples taken at the time of embryo transfer (Table 2). Given that MEA was measured in PBMC, hematology parameters were evaluated to account for the animal's immune status, which could influence MEA outcomes. All cows enrolled as potential recipients were deemed healthy based on the absence of clinical illnesses such as mastitis, lameness, or reproductive diseases. Hematology results were defined by pregnancy outcome confirmed through 90 DCC. Hematological values were not included for one pregnant animal due to poor blood sample quality and values that were beyond 3 SD. Hematology parameters were compared by pregnancy outcome, embryo assigned, and relative to clinically normal reference ranges. Reference ranges were obtained from the manufacturer of the HemaVet analyzer (Erba Diagnostics, Oxford, CT) and used to perform hematological assessments.

Recipients were in the normal reference ranges for all hematological parameters except for neutrophil count, which was slightly higher than the reference range (Table 2). The neutrophil count was not different between embryo assigned (*P* = 0.71). A slightly elevated neutrophil count could suggest a mild inflammatory response, subclinical infection, or stress. Somatic cell count data were collected monthly through routine milk testing on the herd. The second and third monthly tests, conducted around the time of embryo transfer, did not indicate evidence of subclinical infection, with the highest SCC recorded among recipients being 102,000 cells/mL. However, higher mean neutrophil concentrations have been found to be significantly different between reproductive states (*P* < 0.001; Subandrio et al., 2000). Progesterone can have an immunosuppressive effect to protect the developing embryo, which is partially foreign or semiallogenic to the maternal immune system (Herath et al., 2006; Cui et al., 2020).

The platelet count was higher in pregnant cows than nonpregnant cows (Table 2; *P* = 0.03). No differences were observed between embryo assignments, except for mean corpuscular volume. Mean corpuscular volume, which reflects the average size of red blood cells, was 0.04 fL greater in pregnant cows compared with those that were nonpregnant, although both values were in the normal reference range. Overall, the immune status was comparable between pregnancy outcome and embryo assignment.

Mitochondrial enzymes in oxidative phosphorylation were analyzed due to their key role in ATP production during cellular respiration. The C-I and C-IV help create the proton gradient, whereas C-V uses that gradient to generate ATP through oxidative phosphorylation. Mitochondrial enzyme activities between pregnant and nonpregnant recipients and embryos A or B are summarized in Table 3. No differences in MEA were observed at the time of transfer between cows that subsequently maintained pregnancy through 90 DCC, and embryo assignment tended to be different for C-I and C-IV (*P* = 0.1). There were no interactions between pregnancy outcome and embryo (*P* = 0.6, *P* = 0.6, *P* = 0.4 for C-I, C-IV, and C-V, respectively), so the interaction term is not shown in Table 3. Ratios between C-I:C-V, C-IV:C-V, and C-I:C-IV were also evaluated but were not different by pregnancy outcome or embryo (*P* > 0.05, data not shown).

The MEA of each complex was associated with the MEA of the other 2 complexes, which is expected due to the inter-relationships between complexes in creating the proton gradient and C-V using that gradient to generate ATP. The observed positive association between C-I and C-IV is supported by their interdependent roles within the electron transport system. As the primary entry point for electrons, C-I initiates electron flow, whereas C-IV serves as the terminal complex, reducing oxygen to water. Niesen et al. (2022) showed that C-I activity varies with proximity to peak DIM. The C-I activity may vary as cows approach or move past peak lactation, reflecting shifts in energy demand and substrate availability, whereas C-IV activity is related to oxidative stress and signaling of apoptosis.

Complex I MEA was positively associated with MCH, which was within normal ranges according to hematology results (Table 2), and is important for the transport of oxygen to mitochondria to serve as the final electron acceptor to form water. Oxidative phosphorylation uses ∼90% of the oxygen that is inhaled, and most of that is carried by hemoglobin, and so MCH could indicate oxygen supply to mitochondria. But oxygen and hydrogen conversion into water happens at C-IV, and MEA of C-IV was 10-fold higher than C-I (Table 3). Because many other factors affect oxidative phosphorylation such as calcium regulation, hypoxia, reactive oxygen species (**ROS**) production and oxygen availability, the lower MEA of C-I and its association with MCH could be due to proton leak at C-I or C-III (or both), ROS formation, or an environment that favors C-III versus NADH sources of electrons (Schottlender et al., 2021; Gałęska et al., 2025). The association of MCH with C-I may be more indicative of the regulation of oxidative phosphorylation process with C-I as the first step, mitochondrial damage, or proton leak rather than oxygen supply to the mitochondria.

Complex IV activity was negatively associated with platelet count, which was at the low end of the normal range in nonpregnant heifers (Table 2). Platelet count has been associated with improved breeding and fertility outcomes as platelets release many growth factors that stimulate embryo development and endometrial cell proliferation (Lange-Consiglio et al., 2015; Sills and Wood, 2023) so low platelet counts of nonpregnant heifers may be why they did not conceive. Similarly to C-I, C-IV MEA was not different with pregnancy status but tended to be lower in recipients given embryo B (Table 3). Although there were equal numbers of pregnancies between embryo A and B (6 each), the lower activity in both C-I and C-IV with embryo B may mean that there were other physiological challenges, such as an immune challenge with embryo B recipients. Immune function has been shown to directly impact fertility, as cows that have disease postpartum have decreased pregnancy rates and more pregnancy losses, both by genotype (Ortega et al., 2017) and phenotype (Santos et al., 2016).

Because embryo was not different in the analyses of prior performance or pregnancy diagnosis of recipients (Table 1) and embryo was not associated with nearly all hematological variables (Table 2), we initially ran the regressions of prediction of C-I, C-IV, and C-V MEA without embryo A versus B. The resulting predictive equations still showed the interdependence between C-I and C-IV, but both were also associated with gDPR, implying that nuclear-encoded genes regulating mitochondrial function are represented to some extent within the genomic markers used for fertility evaluations. A possible genomic marker for fertility that is associated with the electron transport system and mitochondrial synthesis is an SNP in the gene COQ9. This SNP is associated with 3% of the variation in gDPR in Holstein cows (Ortega et al., 2017). COQ9 is expressed in endometrium, oocyte, and preimplantation embryo and involved in the synthesis of COQ10 which is associated with C-I and C-III (Hidalgo-Gutiérrez et al., 2021) and related to reduced proton leak in oocytes before the blastocyst stage (Ortega et al., 2017). Energy status of the donor cow is important for the development of good-quality oocytes and is important for the recipient to provide a good uterine environment for the embryo (Wathes et al., 2012; Kölle et al., 2020). Reduced activity could be associated with reduced ROS production in C-I and C-III, which is critical to prevent damage to mitochondrial DNA and the electron transport system prior. The tradeoff between gDPR or embryo source in the C-I and C-IV regression equations suggests that embryo source may have represented 2 different SNP for the gene COQ9. These findings highlight the potential practical value of selecting for fertility and examining its relationship with mitochondrial function. Genomic trait values such as gDPR may reflect both reproductive performance and underlying energy metabolism important for cow health and longevity, even though MEA was not linked to pregnancy outcome in this study.

Complex V activity was negatively associated with lymphocytes and gDWP with coefficients of −0.014 and −0.000041, respectively, indicating that higher lymphocytes and gDWP were associated with lower C-V activity. An elevated lymphocyte count could suggest a mild inflammatory response, subclinical infection, or stress. Because enzyme activity was measured in PBMC, this may reflect shifts in the metabolic state or composition of immune cells rather than systemic energy availability. It would be expected that C-V and gDWP would be positively associated because in our previous research C-V activity was higher in higher milk producing cows and was associated with higher gaining calves (Niesen and Rossow, 2019; Niesen et al., 2022) and higher milk production should be associated with more gDWP. Both gDWP and C-I were negatively associated with C-V in this study, indicating that there may have been proton leak or other factors that were causing C-I and gDWP to be negatively associated with C-V activity.
